# Impact of Lung-Related Polygenic Risk Scores on Chronic Obstructive Pulmonary Disease Risk and Their Interaction with w-3 Fatty Acid Intake in Middle-Aged and Elderly Individuals

**DOI:** 10.3390/nu15133062

**Published:** 2023-07-07

**Authors:** Ki-Song Kim, Sunmin Park

**Affiliations:** 1 Department of Physical Therapy, Institute of Basic Science, Hoseo University, Asan 31499, Republic of Korea; kskim68@hoseo.edu; 2 Department of Food and Nutrition, Obesity/Diabetes Research Center, Hoseo University, Asan 31499, Republic of Korea

**Keywords:** lung structure, lung function, polygenic risk score, spirometry, inflammation, chronic obstructive pulmonary disease

## Abstract

Chronic obstructive pulmonary disease (COPD) is a complex, progressive respiratory disorder with persistent airflow limitation and tissue destruction. We aimed to explore the genetic impact of COPD and its interaction with nutrient intake in 8840 middle-aged and elderly individuals from the Ansan/Ansung cohorts. Participants were diagnosed with COPD if the ratio of forced expiratory volume in 1 s (FEV1) to forced vital capacity (FVC) was less than 0.7 using spirometry, and if they were previously diagnosed with COPD by a physician. Genome-wide association studies (GWAS) were performed to screen for genetic variants associated with COPD risk. Among them, we selected the genetic variants that exhibited interactions using the generalized multifactor dimensionality reduction (GMDR) method. The polygenic risk score (PRS) was computed by summing the number of risk alleles in the SNP-SNP interaction models that adhered to specific rules. Subsequently, participants were categorized into low-PRS, medium-PRS, and high-PRS groups. The participants with COPD exhibited significantly lower FEV1/FVC ratios (0.64) than those without COPD (0.82). It was positively associated with inflammation markers (serum C-reactive protein and white blood cell levels). A higher proportion of COPD participants were smokers and engaged in regular exercise. The 5-SNP model consisted of *FAM13A*_rs1585258, *CAV1*_rs1997571, *CPD*_rs719601, *PEPD*_rs10405598, and *ITGA1*_rs889294, and showed a significant association with COPD risk (*p* < 0.001). Participants in the high-PRS group of this model had a 2.2-fold higher risk of COPD than those in the low-PRS group after adjusting for covariates. The PRS interacted with w-3 fatty acid intake and exercise, thus influencing the risk of COPD. There was an increase in COPD incidence among individuals with a higher PRS, particularly those with low consumption of w-3 fatty acid and engaged in high levels of exercise. In conclusion, adults with a high-PRS are susceptible to COPD risk, and w-3 fatty acid intake and exercise may impact the risk of developing COPD, potentially applying to formulate precision medicines to prevent COPD.

## 1. Introduction

Chronic obstructive pulmonary disease (COPD) is a complex and progressive respiratory disorder characterized by persistent reduction in airflow through the lung and accompanied by irreversible lung damage [1]. The presence of the persistent airflow limitation measured as a ratio of forced expiratory volume in 1 s (FEV1) to forced vital capacity (FVC) of <0.7 confirms the diagnosis of COPD in the Global Initiative for Chronic Obstructive Lung Disease (GOLD) guidelines based on the spirometric method [2]. According to the GOLD definition, in 2019, the global prevalence of chronic obstructive pulmonary disease (COPD) among individuals aged 30–79 years was estimated to be 10.3% (95% CI = 8.2–12.8%) [3]. While the incidence rates of COPD vary depending on demographic factors such as age, gender, residence area, and socioeconomic status [4], it remains one of the global health issues. Although long-term cigarette smoking is the primary COPD risk factor [5], environmental elements such as air pollution, indoor and outdoor exposure to pollutants, occupational dust, fumes, and respiratory infections can contribute significantly to COPD incidence [6]. Other lifestyle-related risk factors, proper exercise, and a healthy diet have also been reported to prevent the development and progression of COPD [7].

Genetic factors have been recognized as significant contributors to the development and progression of COPD. In recent years, substantial progress has been made in comprehending the genetic basis of COPD. The specific genes involved in COPD are α1-antitrypsin (*SERPINA1*), serine protease inhibitor-2 (*SERPINE2*), *CHRNA3/5* encoding nicotinic acetylcholine receptor subunits, superoxide dismutase-3 (*SOD3*), and transforming growth factor-β1 (*TGF-β1*) [8,9,10,11]. Genome-wide association studies (GWAS) have identified numerous genetic variants associated with COPD susceptibility, severity, and response to treatment in different ethnicities, including European ancestries [8,12,13]. Genetic polymorphisms of *SERPINE2* (rs16865421), *CHRNA3/5* (rs8034191 and rs1051730), and family with sequence similarity 13 member A (*FAM13A*) rs1903003, rs7671167, and rs1964516 are linked to COPD risk in the Caucasians [8,9,10,11]. Matrix metallopeptidase 12 (*MMP12*; rs626750), superoxide dismutase-3 (*SOD3*; rs1799895 and rs699473), and transforming growth factor-β1 (*TGF-β1*; rs10429950) are related to COPD risk in diverse ethnicities [12,14]. Genetic predisposition plays a role in determining an individual’s susceptibility to COPD and the underlying mechanisms and pathways. The genetic predisposition is associated with lung development and function, protease–antiprotease imbalance, response to environmental insults (such as pollution and cigarette smoke via the antioxidant system), anti-inflammatory response, immunity, and lung tissue remodeling and repair [12,15]. These findings have shed light on preventing its development at an early age by genetic screening for COPD risk.

Interactions between genetic variants and environmental exposures can modify the risk and severity of COPD, leading to inter-individual variations in disease susceptibility and progression [16]. Genetic variants may influence an individual’s ability to metabolize and eliminate toxins present in cigarette smoke, occupational hazard, and pollution, modulating the impact of smoking on lung health [17]. Among the several lifestyle factors linked to COPD risk, the maximum number of studies are on the interaction between smoking and COPD-related genetic variants, such as rs10193706 near testis expressed 41 (*TEX41*), contributing to COPD risk in UK Biobank data and rs1051730, rs16969968, and rs1799895 polymorphisms of glutathione S-transferase Mu-1 (*GSTM1*) interact with smoking, contributing to COPD risk in Mongolians [14,18]. However, such studies on the interaction between genetic and lifestyle factors on COPD risk are limited.

The Ansan/Ansung cohort, a well-characterized population-based study conducted in South Korea, provided a unique opportunity to explore the genetic variants linked to COPD and their interactions with lifestyle factors [19,20]. This cohort consisted of a relatively large sample size (*n* = 8840) of individuals with extensive phenotypic and genotypic data, making it an ideal resource for conducting genetic studies. The present study aimed to comprehensively investigate the genetic variants associated with COPD in the Ansan/Ansung cohort and evaluate their interactions with lifestyle factors. Clarifying the function of genetic variants and their interactions with lifestyle factors could contribute to advancing our knowledge of COPD pathogenesis and assist in identifying new avenues for formulating personalized prevention and treatment measures.

## 2. Methods

### 2.1. Participants

The Korea National Institute of Health initiated the Korean Genome and Epidemiology Study (KoGES), a prospective study focused on middle-aged and elderly Korean individuals in 2001 [21]. After completing initial assessments, individuals aged 40–69 years living in either the urban community of Ansan or the rural community of Ansung were chosen for genetic testing. This testing was conducted as part of the Korea Association Resource, which involved a comprehensive genome-wide genotyping of participants (*n* = 8840). The study protocol of KoGES received approval from the institutional review board of the Korea National Institute of Health, and all participants provided written informed consent. Furthermore, the study was approved by the review board of Hoseo University (1041231-150811-HR-034-01).

### 2.2. Demographic and Anthropometric Measurement and Lifestyle History 

During the health interview, various information was collected, including age, education, income, medical history, smoking status, coffee and caffeine consumption, alcohol intake, and physical activity. Standardized procedures described in previous studies [20] were used to obtain anthropometric measurements, such as height, body weight, waist circumference, and body fat. The body mass index (BMI) was calculated by dividing weight (in kilograms) by the square of height (in meters). Body fat was measured using tetrapolar bioelectrical impedance analysis (Inbody 3.0, Biospace, Seoul, Republic of Korea). Education level was divided into three categories: individuals with less than a high school education, high school graduates, and college degrees or higher.

Personal stress levels were assessed using a total of 40 questionnaires, which were categorized into stress-related behaviors, psychological signs, physical signs, and behavioral signs. Each category consisted of ten questionnaires. Each questionnaire utilized a “yes” response to indicate high stress or a “no” response to indicate no stress. These responses were assigned values of 1 or 0, respectively, to calculate the stress scores by summing the individual scores. The questionnaires can be found in Supplementary Table S1. Sleep-related behaviors were also surveyed, including insomnia, snoring, and awakening due to difficulty breathing during sleep. Additionally, experiences of sputum and cough were determined.

Coffee and alcohol intakes were assessed based on information collected about the frequency of consuming one or more servings of coffee or alcohol per day during the month preceding the interview. The specific types of coffee and alcohol consumed were also considered to estimate the intake. The intake was measured by multiplying the volume consumed in a single instance by the reported frequency. The collected data categorized participants into three groups according to their coffee consumption: those who consumed no coffee or less than three cups per week, moderate coffee consumers with weekly 3–10 cups, and heavy coffee consumers with >10 cups per week. Caffeine intake was determined based on coffee, tea, chocolate, and cola consumption. The calculated caffeine intake was further classified into three groups: less than 60 mg, 60–220 mg, and more than 220 mg daily. Participants were grouped into four categories based on their average daily alcohol intake: non-drinkers, light drinkers (1–15 g), moderate drinkers (16–30 g), and heavy drinkers (>30 g). Smoking status was assessed and divided into three categories: current, past, and never-smokers. The cutoff for current and past smokers was set at having smoked more than 100 cigarettes in their lifetime and within the last 6 months.

Regular physical activity levels were determined considering the duration and intensity of exercise. The moderate exercise included playing slow swimming, volleyball, doubles tennis, and occupational or recreational activities involving light objects performed regularly for more than 30 min at a time, at least five times per week. The vigorous exercise included activities such as running, climbing, rapid cycling, rapid swimming, football, basketball, singles tennis, jumping rope, and squash, as well as occupational or recreational activities involving heavy objects. These activities were performed for more than 20 min at a time, at least three times per week.

### 2.3. Biochemical Assessment

Serum high-sensitive C-reactive protein (hs-CRP) concentrations were determined using a high-sensitive ELISA kit (Thermofisher, Waltham, MA, USA). White blood cell (WBC) counts were assessed using EDTA-treated blood. Serum lead (Pb) and cadmium (Cd) concentrations were quantified using Inductively Coupled Plasma-Atomic Emission Spectrometers (ICP-AES; Thermofisher, Waltham, MA, USA).

### 2.4. Food and Nutrient Intake Assessment by a Semi-Quantitative Food Frequency Questionnaire (SQFFQ)

Daily dietary intake was assessed with an SQFFQ specifically designed and validated for Korean adults in the KoGES study. The SQFFQ aimed to gather information on the participants’ consumption of various food items. A total of 8840 participants completed the SQFFQ, which included questions on the consumption of 103 food items [22]. These food items were then analyzed based on the macro- and micro-nutrients in the Korean food composition table. The accuracy of the SQFFQ was validated by comparing it with 3-day food records collected during four different seasons, and this validation process helped ensure the reliability of the questionnaire [22]. The average daily nutrient intake from the SQFFQ results was determined using the computer-aided nutritional analysis program (CAN Pro) 3.0. CAN Pro 3.0 utilizes a nutrient database developed by the Korean Nutrition Society (Seoul, Republic of Korea) to calculate nutrient values.

### 2.5. Definition of COPD

Participants with a ratio of FEV1 to FVC (FEV1/FVC) < 0.7 and a previous history of COPD diagnosis by a physician were considered to have COPD [2]. The COPD and Healthy groups included 875 and 7965 subjects, respectively.

### 2.6. Genotyping and Quality Control

The genotype data were provided by the Center for Genome Science at the Korea National Institute of Health. Genomic DNA was extracted from whole blood samples, and genotyping was carried out using the Affymetrix Genome-Wide Human SNP Array 5.0 (Affymetrix, Santa Clara, CA, USA). The genotyping data underwent assessment using the Mahalanobis Distance Genotyping Algorithm with Bayesian Robust Linear Modeling to ensure quality and accuracy of the genotyping [23]. Genotypes that did not meet specific criteria were excluded from the analysis. The exclusion criteria are those with low genotyping accuracies (<98%), excessive heterozygosity (>30%), high rates of missing genotype calls (≥4%), gender biases, or deviations from the Hardy–Weinberg equilibrium (HWE) (*p* < 0.05) [23].

### 2.7. Selection of Interacting Genetic Variants for COPD Risk by GWAS Followed by a Generalized Multifactor Dimensionality Reduction (GMDR) Method

**Figure 1:**
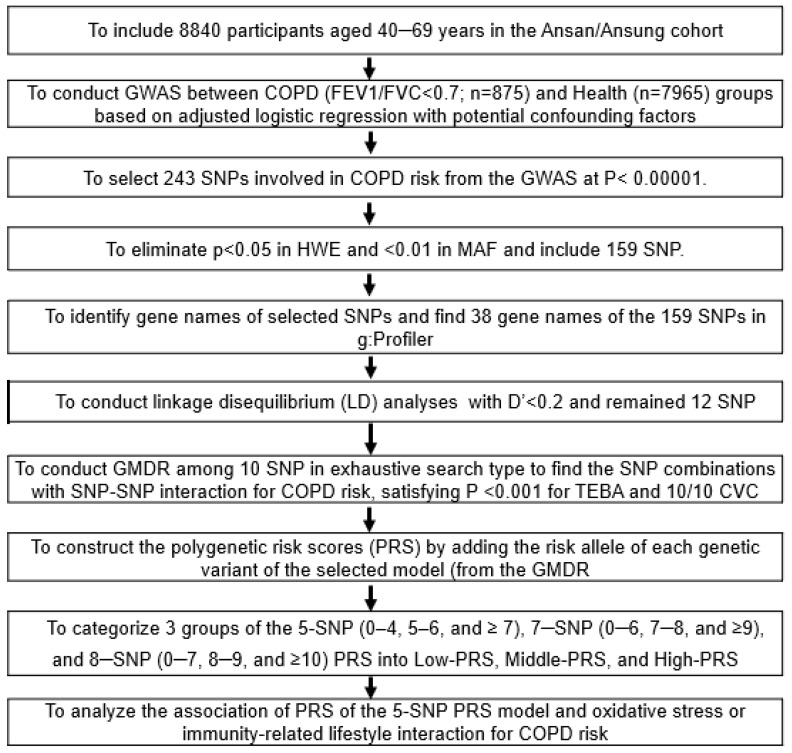
A flowchart illustrating the process of genetic variant selection, SNP–SNP interaction modeling, construction of polygenic risk scores (PRS), and the procedure for assessing the interaction between PRS and lifestyle factors for chronic obstructive pulmonary disease (COPD) risk.

Figure 1 illustrates the process of genetic variant selection, SNP–SNP interaction modeling, and the procedure for assessing the interaction between polygenic risk scores (PRS) and lifestyle factors. GWAS was performed to investigate the genetic impact on COPD in the Ansan/Ansung cohort. The analysis was adjusted for age, gender, city residence area, BMI, job experience in companies dealing with chemicals or with a dusty work environment, energy and alcohol intake, physical exercise, and smoking were assessed using PLINK (http://pngu.mgh.harvard.edu/~purcell/plink, accessed on 4 December 2022). Manhattan and Quantile–Quantile (Q–Q) plots were employed to validate the suitability of the genetic variants for COPD. The lambda value of the Q–Q plot indicated the extent of inflation in test statistics due to factors such as population stratification or sample relatedness. When it was close to 1, the genotypes by GWAS were appropriate. Single nucleotide polymorphisms (SNPs) displaying high linkage disequilibrium (LD) (D′ < 0.2) were excluded from further analysis to avoid redundancy in the genetic information. Haploview 4.2 in PLINK was used to ensure that SNPs with similar genetic information were not considered [24]. GMDR is a non-parametric genetic model designed to detect and characterize nonlinear interactions among discrete genetic attributes. The GMDR method was employed to explore genetic variants to interact. Using the results of the GMDR analysis, the best models of interactions between genetic variants associated with COPD risk were identified to enhance our understanding of the genetic impact. Criteria were established for selecting the optimal model to satisfy a significant *p*-value (*p* < 0.05) for the sign test of trained balance accuracy (TRBA) and test balance accuracy (TEBA), as well as achieving a cross-validation consistency (CVC) score of 9 or 10 out of 10 [24]. The polygenic risk score (PRS) was calculated by summing the number of the risk alleles in the genetic variants included in each selected model. The PRS was computed by adding the number of risk alleles present in the genetic variants encompassed by each selected model. Participants were then categorized into three groups: Low-PRS, Middle-PRS, and High-PRS, based on their PRS values in each PRS model. The Low-PRS group consisted of individuals with fewer than one copy of the risk allele across all genetic variants in the model, while the High-PRS group comprised individuals with more than two genetic variants showing homozygotes for the risk alleles.

### 2.8. Statistical Analysis

Statistical analysis was conducted using SAS (version 9.3; SAS Institute, Cary, NC, USA). The categorical variables of the participants, including gender, income and education levels, smoking status, and others, were examined by determining the frequency distributions based on the tertiles of 5-SNP PRS categories, namely, low, medium, and high groups (≤4, 5–6, and ≥7, respectively). The Chi-squared test was applied to evaluate the frequency distributions of the categorical variables. Additionally, a one-way analysis of variance (ANOVA) was conducted to compare the PRS categories across continuous variables such as age, BMI, energy intake, and others. The statistical differences between Healthy and COPD groups in FEV1 and FEV1/FVC according to age groups were determined with two-way ANOVA.

The association between anthropometric, biochemical, and genetic parameters and COPD risk was conducted using adjusted logistic regression analysis. It was examined with two different sets of potential confounders, referred to as covariate sets 1 and 2. The covariate set 1 had adjustments for the residence area, gender, age, and BMI. The covariate set 2 included adjustments for covariates in covariate set 1 plus job history in a chemical and companies dealing with chemicals or a dusty work environment, drinking, smoking, total physical activity, medication for asthma, and energy intake. Adjusted odds ratios (ORs) and 95% confidence intervals (CIs) were calculated using the low-PRS group as the reference category.

The potential interaction between PRS and lifestyle factors influencing COPD risk was examined using a multivariate general linear model (GLM) with PRS, lifestyle factors, and covariates from covariate set 2. If a significant interaction was found in the multivariate GML model with main effects and their interaction term, an adjusted logistic regression analysis was conducted in two groups based on known cutoff values such as dietary reference intake (DRI) of each nutrient, or, if not available, using ≤25th percentile as the cutoff for the low level. The remaining values were classified as high. The specific cutoff values for each parameter can be found in the legend of each table. Based on the classification criteria, participants were then categorized into high and low groups. Statistical significance was set at a *p*-value of ≤0.05.

## 3. Results

### 3.1. Demographic, Anthropometric, and Biochemical Characteristics of Participants

The percentage of participants with COPD was about 9.9% (*n* = 875). FEV1 was significantly lower in the COPD group than in the Healthy group, but was not significantly different by age group (Table 1). The participants in the COPD and Healthy groups had an FEV1/FVC ratio of 0.64 and 0.82, respectively, and those with COPD had lower FEV1 percentages based on the predicted value than those in the Healthy group (Table 1). The FEV1/FVC ratio decreased with the elderly group, unlike FEV1 (Table 1). Participants in the COPD group were older than those in the Healthy group, and there were a higher number of males in the COPD group than in the Healthy group. The COPD group had a lower number of participants residing in cities than the Healthy group, and an inverse association was observed between residence in a city and the incidence of COPD. The percentage of participants with COPD did not vary according to education or employment in jobs requiring exposure to chemicals or dust (Table 1). No significant differences were observed in stress-related behaviors, psychological signs, physical signs, and behavioral signs of stress between the COPD and Healthy groups, as shown in Table 1. Sleep disorders, including insomnia and snoring, did not exhibit any notable differences between the two groups. However, experiencing awakening due to difficulty breathing during sleep was positively associated with COPD, with a 1.81-fold increased risk compared to the Healthy group. Furthermore, the occurrence of sputum was 1.3 times higher in the COPD group than in the Healthy group.

Anthropometric measurements were different between the COPD and Healthy groups, with height, BMI, and waist circumferences being lower in the COPD group than in the Healthy group. The white blood cell (WBC) count and serum C-reactive protein (CRP) concentrations were higher in the COPD group than in the Healthy group and were positively linked to COPD (Table 1). Serum Pb concentrations, but not Cd, were higher in the COPD group than in the Healthy group by 1.9 times. It indicated that participants with COPD had elevated inflammatory states and serum Pb concentrations.

The COPD group exhibited a significantly higher percentage of asthma participants than the Healthy group. The incidence of asthma showed a 2.87-fold higher positive association with COPD, indicating a strong link between these two respiratory conditions (Table 1). Allergies were not significantly associated with COPD (Table 1).

### 3.2. Nutrient Intake and Lifestyle Factors

The daily energy intake of participants with COPD based on the DRI did not differ between the COPD and Healthy groups. The consumption of macronutrients, including carbohydrates, proteins, total fats, and w-3 and w-6 fatty acids, did not vary between the two groups (Table 2). Micronutrient intake, including total polyphenols, total flavonoids, vitamin C, vitamin D, vitamin E, selenium (Se), and calcium (Ca), did not vary between the COPD and Healthy groups. Alcohol intake also did not vary between the two groups. Surprisingly, the COPD group exhibited a higher percentage of individuals engaging in moderate-intensity exercise than the Healthy group, as shown in Table 2. It suggested that despite engaging in moderate-intensity exercise, individuals in the COPD group still had a higher risk of developing COPD. The percentage of smokers was much higher in the COPD group than in the Healthy group, and smoking was positively associated with COPD risk (Table 2).

### 3.3. Selection of the Genetic Variants Linked to COPD

**Figure 2:**
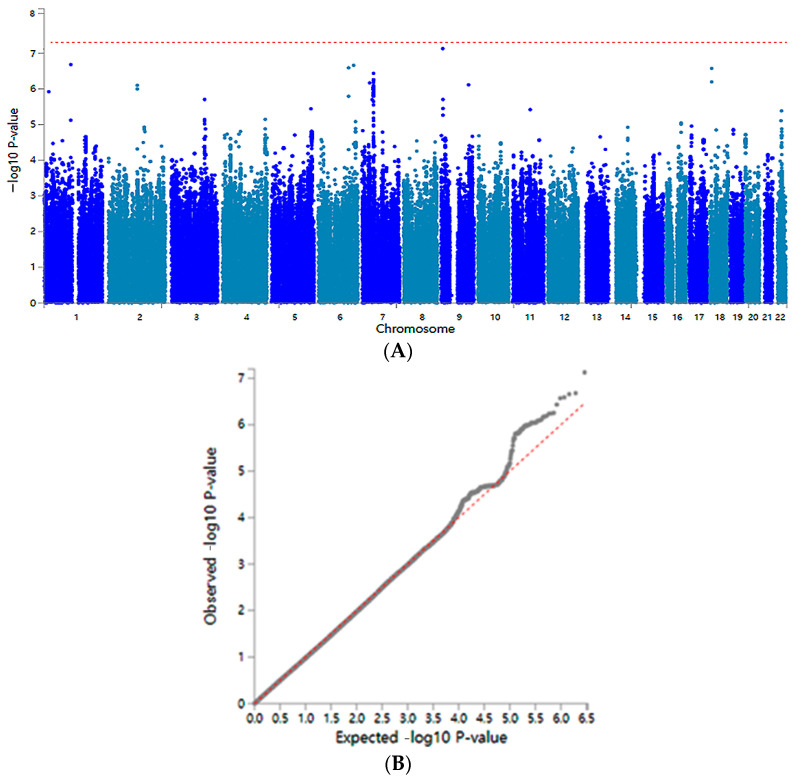
Genetic variant distribution for COPD risk in a genome-wide association study. (**A**) Manhattan plot of the *p*-value of genetic variants. The red dotted line indicates the *p*-value of the cutoff of genetic variants for the COPD risk (**B**) Q–Q plot of observed and expected *p*-values. The red dotted line indicated the calculated observed and expected *p*-value. It indicates the perfect matching between observed and expected *p*-values.

The association of genetic variants with COPD is presented with their statistical differences in a Manhattan plot (Figure 2A). In the Q-Q plot, the quantile distribution between the observed and expected statistical difference and the lambda value was 1.085. Thus, the genetic variants had no bias or inflation in the GWAS of COPD (Figure 2B).

Genetic variants linked to COPD were selected from the results of GWAS at *p* = 5 × 10^−5^, which was higher than the *p*-value with Bonferroni correction since more than 50% of the genetic variants showed an LD relation. Genetic variants that did not meet the inclusion criteria were excluded from further analysis to ensure that only the most relevant and independent genetic variants were considered for the subsequent analyses. Among the genes for the selected genetic variants, those with the same pathway were selected to examine genetic variant interactions using GMDR. Ten genetic variants met the HWE (*p* > 0.05). The ten genetic variants included in the GMDR were zinc finger protein 385D (*ZNF385D*), family with sequence similarity 13 member A (*FAM13A*), integrin subunit-a 1 (*ITGA1*), ATP binding cassette subfamily A member 13 (*ABCA13*), caveolin 1 (*CAV1*), protein tyrosine phosphatase receptor type delta (*PTPRD*), neurotrimin *(NTM)*, carboxypeptidase D (*CPD*), microtubule crosslinking factor 1 (*MTCL1*), and peptidase D (*PEPD*) (Table 3). The alleles *FAM13A*_rs1585258, *ABCA13*_rs4145714, and *PTPRD_*rs10959052 were inversely associated with COPD, and seven other genetic variants showed a positive association with COPD.

### 3.4. Model for SNP-SNP Interactions and the Association of PRS with COPD Risk

In the GMDR analysis, the optimal interaction model was determined from the 10 genetic variants listed in Table 3. Interaction models that satisfied a significance threshold of *p* = 0.001 for the sign test and achieved a CVC score of 10/10 were considered. Based on these criteria, the genetic variants numbered 5, 7, 8, and 10 were included in the optimal interaction model (Supplementary Table S2).

**Figure 3:**
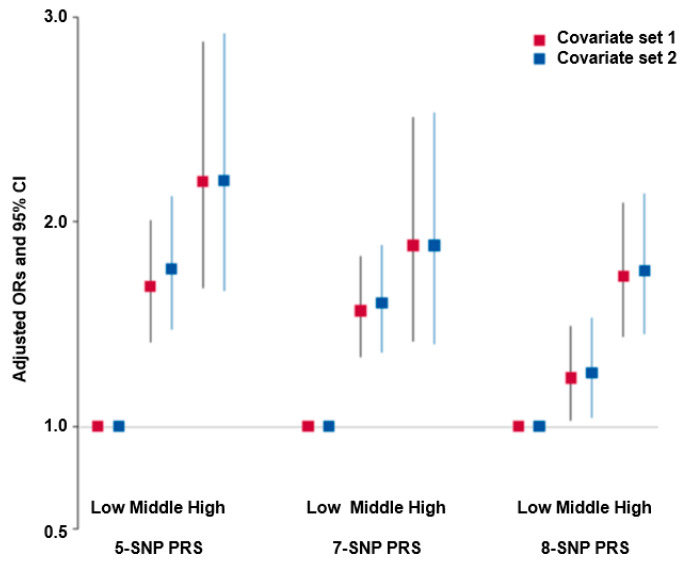
Adjusted odds ratio (ORs) and 95% confidence intervals (CI) of the 5-, 7-, and 8-SNP PRS for COPD risk. PRS was generated with the sum of the number of risk alleles in each SNP and was classified into low-PRS, middle-PRS, and high-PRS according to the ranges of ≤4, 5–6, and ≥7 in the five-SNP model, 0–6, 7–8, and ≥9 in the seven-SNP model, and 0–7, 8–9, and ≥10, respectively. The covariates of set 1 were age, gender, body mass index, education, income, and residence area. Group 2 included those in set 1 plus energy intake, work-related chemicals and dust, asthma medication, exercise, alcohol drinking, and smoking.

The 5-SNP model included *FAM13A*_rs1585258, *CAV1*_rs1997571, *CPD*_rs719601, *PEPD*_rs10405598, and *ITGA1*_rs889294. Participants in the high-PRS group of the 5-SNP model had 2.196- and 2.202-times higher risks of COPD than those in the low-PRS group after adjusting for covariate sets 1 and 2, respectively (Figure 3). Covariate set 1 included potential confounders such as age, residence area, gender, and BMI in adjusted logistic regression analysis, and covariate set 2 contained those in covariate set 1 plus job factors, smoking, coffee, alcohol, physical activity, and energy intake for model 2. The 7-SNP and 8-SNP PRS were also positively associated with COPD risk. However, the adjusted ORs for the 7-SNP and 8-SNP PRS models were much smaller than that for the 5-SNP PRS (Figure 3). Therefore, the 5-SNP PRS was used to analyze the interaction with lifestyle factors.

### 3.5. Gene Expression of Genetic Variants in Different Tissues

The minor allele of *FAM13A_*rs1585258 was associated with a weak decrease in *FAM13A* expression in the lung and tibial nerve (β = −0.11 and −0.087) (Table 4). The minor allele of *ITGA1*_rs889294 was linked to a weak increase in *ITGA1* expression in the subcutaneous and visceral adipose tissue, skeletal muscle, tibial nerve, and left ventricle of the heart (β = 0.11–0.17). However, *CAV1*_rs1997571 with the minor allele showed a slight decrease in the CAV1 expression in the tibial nerve but a moderate increase in the atrial appendage of the heart (β = −0.78 and 0.13). *CPD*_rs719601 with a minor allele was inversely associated with CPD expression in the skeletal muscle and lung (β = −0.089 and −0.054) (Table 4). The minor allele of *PEPD*_rs17569 was linked to a modest decrease in the *PEPD* gene in the lung and various other tissues such as the skeletal muscle, adipose tissues, cortex of the brain, tibial nerve, and atrial appendage and left ventricle of the heart (β= −0.2–−0.45) (Table 4). Therefore, the majority of the selected genetic variants exerted control over gene function through their impact on gene expression, which is influenced by the specific allele types in individuals. The different alleles of these genetic variants likely contribute to variations in gene activity and subsequently influence the overall functionality of the genes involved.

### 3.6. Interaction of PRS and Lifestyles Related to Oxidative Stress and Inflammation

**Figure 4:**
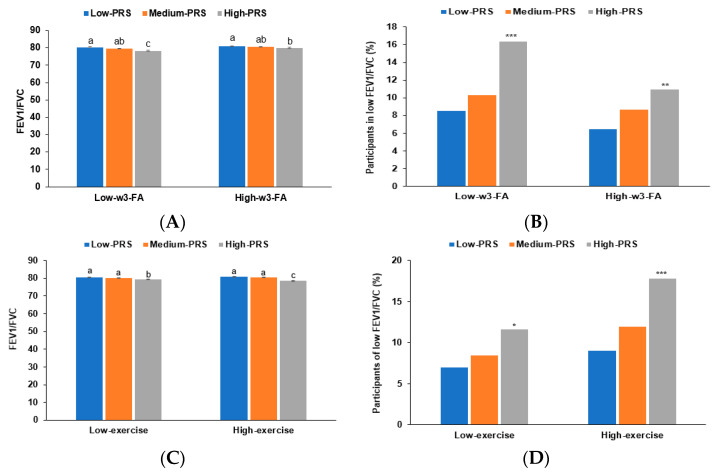
FEV1/FVC of participants with low, middle, or high polygenic risk scores (PRSs) as determined using the 5-SNP model according to w-3 fatty acid intake and exercise. (**A**) Adjusted means and standard errors of forced expiratory volume in 1 s (FEV1)/forced vital capacity (FVC) in the PRS categories by w3-fatty acid intake (a cutoff value: 0.7 En%). (**B**) Incidence of COPD according to the PRS categories by w3-fatty acid intake (a cutoff value: 0.7 En%). (**C**) Adjusted means and standard errors of the FEV1/FVC in the PRS categories by moderate-intensity exercise (a cutoff value: moderate-intensity exercise for 150 min/week). (**D**) Incidence of COPD according to the PRS categories in low and high exercise categorized by moderate-intensity exercise (a cutoff value: moderate-intensity exercise for 150 min/week). PRS was generated as the sum of the number of risk alleles in each SNP, and it was classified as low-PRS, middle-PRS, and high-PRS according to the range ≤4, 5–6, and ≥7, respectively, in the five-SNP model. Covariates included age, gender, education, income, energy intake (percentage of estimated energy requirement), job/occupation with exposure to dust/chemicals, residence area, regular exercise, alcohol intake, and smoking status. * Significantly different between low and high-PRSs at *p* < 0.05, ** at *p* < 0.01, and *** at *p* < 0.001. ^a,b,c^ Different letters on the bar indicated significant differences among the groups in Tukey’s test at *p* < 0.05.

The PRS had no interaction with the daily energy and nutrient intake, alcohol consumption, and smoking status to influence COPD risk. It interacted only with w-3 fatty acid intake and physical exercise to affect COPD risk. The adjusted OR for the PRS was much higher in the low w-3 fatty acid intake (OR = 2.291; 95% CI = 1.642–3.197) than in the high w-3 fatty acid intake group (OR = 1.445; 95% CI = 1.076–1.941; Table 5). The FEV1/FVC lowered in the high-PRS group consuming low w-3 fatty acids than those consuming high w-3 fatty acids (Figure 4A). The COPD incidence was higher with high-PRS in the low w-3 fatty acid intake group than that in the high w-3 fatty acid intake (Figure 4B). However, the adjusted OR was much higher in the high exercise group (2.368) than in the low exercise group (1.555). The decrease in FEV1/FVC with high-PRS was greater in the high-exercise group than in the low-exercise group (Figure 4C). The percentage of the participants with high-PRS was much higher in the high-exercise group than in the low-exercise group (Figure 4D).

## 4. Discussion

The present study investigated the genetic impact of the PRS on COPD and its interaction with lifestyle factors in a large cohort of middle-aged and elderly persons from the Ansan/Ansung cohorts. Our findings provided several important insights into the genetic basis of COPD susceptibility and its relationship with lifestyle choices. These genetic variants have been previously implicated in COPD pathogenesis through various mechanisms, including inflammation, extracellular matrix remodeling, and lung development. We evaluated the genetic contribution to COPD risk by identifying significant genetic variants and calculating the PRS. We highlight the utility of the PRS in assessing individual susceptibility to COPD and the potential function of w-3 fatty acid intake and exercise in modifying the impact of genetic risk. These findings have implications for precision medicine approaches to prevent and manage COPD, opening avenues for further research into targeted interventions and personalized strategies for at-risk individuals.

A significant proportion of COPD cases, estimated to be up to 85%, remain undiagnosed, indicating a potential unmet need in identifying and managing these individuals [25]. Recent studies have also examined patients with respiratory symptoms but normal lung function. In this study, the participants who did not diagnose but had low FEV1/FVC were included in the COPD group, and they were classified into mild COPD according to GOLD1. Comparative analysis revealed significantly higher proportions of patients with sputum production, cough, and awakening due to breathlessness in the COPD group compared to healthy individuals. However, no significant differences were observed in the scores of psychological signs, physical signs, and behavioral signs, as well as stress-related behaviors, when compared to the Healthy group. These findings suggest that COPD in this study was associated with airway obstruction caused by emphysema, asthma, or bronchitis, rather than stress or allergies.

COPD is influenced by various risk factors, including age, gender, genetics, and lifestyle-related factors such as smoking, air pollution, occupational exposures, socioeconomic status, immune response, inflammation, and BMI [26]. Among these factors, smoking is the primary and most significant risk factor for COPD as it is associated with pulmonary vascular endothelial cell apoptosis in COPD pathogenesis [27]. In the present study, current smoking was found to be positively associated with COPD, while former smoking did not show a significant association. These findings align with previous research highlighting a higher incidence of COPD among men due to higher smoking rates. However, it is important to note that women who smoke have a 50% higher likelihood of developing COPD than men, and smoking has a more pronounced impact on respiratory function decline in women than men [28]. With the increasing prevalence of smoking among women, there is a concern that the incidence of COPD in women may rise accordingly.

Occupational dust and chemical fumes are recognized as risk factors for COPD. Exposure to workplace pollutants, primarily inorganic dust, can initiate airway damage and inflammation, which are primary mechanisms in the development of COPD [29]. However, diagnosing occupational COPD remains challenging, primarily due to the difficulty in assessing the specific occupational components contributing to COPD in clinical settings, particularly when other predominant risk factors, such as smoking, are present [29]. In the present study, no significant association was found between work-related exposure to chemicals and dust and the incidence of COPD. The relationship between BMI and COPD is still a matter of debate. A meta-analysis of 30 studies with a total of 1,578,449 participants revealed that the underweight group had a pooled adjusted OR of 1.96 (95% CI = 1.78–2.17) for COPD, while the overweight group had a lower risk with an adjusted OR of 0.80 (95% CI = 0.73–0.87), and the obese group had an adjusted OR of 0.86 (95% CI = 0.73–1.02), compared to the normal-weight group [30]. These findings suggest that being underweight may increase the risk of COPD, while being overweight may decrease it. In line with these previous results, the present study demonstrated a similar trend, where individuals with COPD had lower BMI and body fat than those without the condition, although the impact was minimal.

Genetic factors are known to play a crucial role in the development and progression of COPD. Among the known genetic risk factors, a deficiency in α1-antitrypsin, an enzyme that protects the lungs from inflammation-induced damage, is well-established [31]. In addition to α1-antitrypsin deficiency, genome-wide association studies (GWAS) have identified numerous genetic variants associated with COPD susceptibility. However, in the present study, no specific test was conducted for α1-antitrypsin deficiency, and the genes encoding α1-antitrypsin (*SERPINA1*) and its related genes (such as *SERPINA3*, *SERPINA6*, and *SERPINA10*) were not found to be associated with COPD. These findings suggest that the genetic association of α1-antitrypsin and its related genes may not have a substantial impact on COPD risk in the participants of this study.

The present study identifies the potential genetic variants of the following genes: *FAM13A*, *CHRNA*, *ADAM33*, *GSTM1*, *TNF-α*, *VDBP*, *HHIP*, and *HMOX1* [12]. These genes are involved in lung development, repair, and function, but their exact role in COPD is not yet fully understood. The genes selected for COPD risk in the present study were shown to be associated with COPD, although only a few genetic variants have been studied. *FAM13A* is reported to be involved in airway epithelium remodeling, contributing to COPD [32]. *ZNF385D* expression was related to COPD risk in an analysis of differentially expressed genes [33]. *ITGA1* was associated with COPD and expressed in the lung [34], as shown in the present study. *ABCA13* is involved in maintaining cellular lipid homeostasis by moving lipids inside the cell and in its plasma membrane, as well as removing lipids from the cells, and its mutation can induce a lipid metabolism disorder. The *ABCA13* mutation may be linked with COPD along with atherosclerosis [35]. CAV1, as the structural protein component of caveolae, plays a crucial role in various cellular processes, including signal transduction. It acts as a scaffolding protein within caveolar membranes, facilitating the concentration, organization, and functional regulation of signaling molecules. Notably, CAV1 deficiency in lung fibroblasts has been found to profoundly impact premature senescence induced by oxidants present in cigarette smoke [36]. The presence of CAV1 mutations has been implicated as a potential link to COPD, particularly when considering its interaction with smoking. The protein expressed by the *PEPD* gene cleaves imidodipeptides containing C-terminal proline or hydroxyproline, catalyzing collagen turnover and matrix remodeling [37]. The *PEPD* mutation is related to COPD [38]. Therefore, the genetic variants for COPD risk were linked to the lung structure, remodeling, and function, but not directly to oxidative stress and inflammation in the cohort.

Diet and nutrient intake are also related to the risk of COPD. Since COPD is linked to oxidative stress and inflammation, the consumption of antioxidants and anti-inflammatory foods has the potential to protect against the development and progression of COPD [39]. Previous studies have shown that COPD patients have higher levels of oxidative stress, indicated by elevated malondialdehyde levels and lower levels of vitamin E compared to controls. It suggests that an adequate intake of antioxidant nutrients may help reduce oxidative stress in COPD patients [40]. However, the present study did not find significant differences in the intake of antioxidants, including polyphenols, flavonoids, vitamins C, E, D, and selenium, between the COPD and Healthy groups.

Exercise is widely recognized as a beneficial factor for improving respiratory function by strengthening the respiratory muscles and enhancing lung efficiency. Regular exercise is recommended as part of the management and treatment of COPD [41]. However, since the present study was cross-sectional, reverse causality could potentially explain the findings. Specifically, a higher proportion of participants with COPD were observed in the regular exercise group compared to the low exercise group. It suggests that individuals with COPD may prioritize exercise to maintain their health and manage respiratory symptoms. Nonetheless, it highlights that exercise alone cannot overcome COPD. Furthermore, the study has limitations in determining whether individuals with COPD focused more on exercise for health maintenance due to concerns about respiratory symptoms compared to healthy individuals. Unfortunately, no relevant questions were available to assess this aspect. It is plausible that healthcare professionals advised individuals with COPD to engage in exercise as part of their management plan, which could have contributed to their higher motivation to participate in exercise activities. Moreover, the exercise type and duration defined in the study, which consisted of moderate-intensity exercise for 150 min per week, may not be optimal for effectively managing COPD risk. Further research is needed to explore and identify the most appropriate exercise regimens that can effectively prevent and alleviate the risk of COPD.

These studies have considered various factors such as antioxidant intake, exercise intensity, duration, and specific recommendations tailored to individuals with COPD. By better understanding the beneficial diet and exercise patterns for COPD prevention and management, we can provide more targeted and practical strategies to improve the outcomes for individuals at risk of or living with COPD. A previous meta-analysis has shown that w-3 fatty acid intake is inversely associated with serum interleukin (IL)-6 concentrations, but not lung function [42]. A higher total w-3 intake is related to improving lung function by reducing oxidative stress and inflammation [43,44]. The present study showed no association of w-3 fatty acid intake with COPD in middle-aged and elderly participants, but w-3 fatty acids interacted with the PRS to influence lung function. It indicates that w-3 fatty acid intake directly affects lung regeneration and repair to improve lung function. A meta-analysis including six randomized clinical trials has demonstrated that w-3 fatty acids can effectively improve respiratory function and promote the recovery of lung injury [45,46]. Furthermore, w-3 fatty intake enhances respiratory function, mainly by reducing low-grade systemic inflammation in COPD patients [47]. Therefore, w-3 intake may not only ameliorate inflammation in COPD, but also improve lung function by interacting with the genetic variants.

This study presents several novel contributions to the understanding of the genetic basis of COPD. Firstly, it explores the PRS associated with lung repair and function, shedding light on the genetic factors influencing COPD development. Additionally, the study investigates the interaction between the PRS and lifestyle factors, specifically moderate physical exercise and omega-3 fatty acid intake, providing insights into the gene-environment interplay in COPD. Furthermore, the study explores the potential application of precision medicine in COPD prevention, highlighting the prospects of personalized approaches to disease management. However, it is important to acknowledge certain limitations of the present study. These include: (1) Cross-sectional studies assess data at a single point, making it difficult to establish the temporal sequence between genetic variants and COPD risk. (2) They cannot establish a cause-and-effect relationship. (3) They are susceptible to reverse causality, wherein the nutrient intake may influence the COPD rather than COPD incidence modulating the nutrient intake. Finally, nutrient intake was measured with an SQFFQ, which can under- or over-estimate the usual intake.

## 5. Conclusions

This study highlights the genetic variants linked to COPD risk. It reveals the interplay between the PRS related to lung generation and function, moderate-intensity exercise, and w-3 fatty acid intake as crucial lifestyle factors influencing COPD development. Individuals with a higher PRS face an increased risk of COPD, particularly when coupled with low w-3 fatty acid intake and high levels of exercise. Notably, for adults with a high PRS, incorporating a diet rich in w-3 fatty acids is crucial, while moderate physical exercise may not significantly improve lung function. These findings emphasize the potential of precision medicine strategies in preventing COPD by taking into consideration individual genetic profiles and lifestyle choices.

## Figures and Tables

**Table 1:** Adjusted means and odds ratio (OR) of demographic, anthropometric, and biochemical parameters between Health and COPD groups.

	Health (*n* = 7965)	COPD (*n* = 875)	Adjusted OR and 95% CI
FEV1 (% predicted)	113.8 ± 0.18	92.5 ± 0.58 ***	2.479 (2.073–2.965)
<55 years	111.3 ± 0.26	93.2 ± 0.76 ***	
55–65 years	114.3 ± 0.40	91.3 ± 0.94	
≥65 years	115.8 ± 0.60	90.2 ± 1.17	
FEV1/FVC ^1^	81.5 ± 0.12	63.9 ± 0.33 ***	2.293 (1.643–3.202)
<55 years	82.0 ± 0.09	64.7 ± 0.29 ***^, +++^	
55–65 year	81.2 ± 0.13	63.3 ± 0.35	
≥65 years	81.0 ± 0.20	63.2 ± 0.44	
Age ^2^	51.2 ± 0.09	52.6 ± 0.29 ***	1.504 (1.228–1.843)
Gender (Male, *n*, %)	3657 (45.9)	525 (60.0) ***	1.503 (1.177–1.918)
Education (Yes, *n*, %)			
<High school	4351 (55.1)	551 (63.6)	1
High school	2477 (31.4)	231 (26.6)	0.854 (0.681–1.069)
>High school	1073 (13.6)	85 (9.8)	0.676 (0.494–0.925)
Area (city, *n*, %)	4281 (53.8)	355 (40.6) ***	0.786 (0.620–0.995)
Job			
Chemical Job (Yes, *n*, %)	565 (7.20)	61 (7.08)	1.124 (0.782–1.616)
Dust Job (Yes, *n*, %)	1450 (18.5)	153 (17.9)	0.808 (0.527–1.238)
Psychological stress (score, ≤2)	2.25 ± 0.02	2.29 ± 0.07	0.952 (0.800–1.133)
Physical stress (score, ≤2)	2.83 ± 0.02	2.80 ± 0.08	1.023 (0.851–1.229)
Behavior stress (score, ≤2)	1.53 ± 0.02	1.53 ± 0.06	0.967 (0.809–1.156)
Stress-related behavior (score, ≤1)	2.11 ± 0.02	2.03 ± 0.07	1.012 (0.848–1.206)
Total stress (score, ≤4)	8.74 ± 0.07	8.64 ± 0.22	0.927 (0.760–1.129)
Insomnia (Yes, *n*, %)	711 (9.77)	146 (10.2)	1.052 (0.833–1.328)
Sleep period (<6 h, *n*, %)	6.74+0.02	6.69 + 0.05	1.161 (0.931–1.449)
Snoring (Yes, *n*, %)	341 (10.6)	528 (9.49)	0.914 (0.783–1.068)
Awaking due to hard breathing (Yes, *n*, %)	761 (9.65)	104 (12.6) **	1.814 (1.285–2.562)
Sputum (Yes, *n*, %)	740 (9.53)	125 (12.9) ***	1.300 (1.019–1.660)
Cough (Yes, *n*, %)	809 (9.69)	53 (14.1) **	1.407 (0.961–2.059)
Height (cm) ^3^	160 ± 0.06	161 ± 0.19 **	1.135 (0.888–1.452)
BMI (Kg/m^2^) ^4^	24.7 ± 0.04	24.3 ± 0.12 **	0.916 (0.776–1.081)
Low BMI (*n*, %)	614 (10.6)	261 (8.52) **	
Body fat (%) ^5^	27.0 ± 0.06	26.5 ± 0.20 *	0.950 (0.752–1.200)
WBC (×10^3^/mL) ^6^	6.58 ± 0.02	6.68 ± 0.06 *	1.267 (1.031–1.555)
Serum CRP (mg/dL) ^7^	0.23 ± 0.01	0.25 ± 0.02 *	1.319 (1.091–1.595)
Serum Pb (μg/mL) ^8^	4.45 ± 0.10	5.06 ± 0.26 *	1.914 (1.116–3.283)
Serum Cd (μg/mL) ^9^	1.09 ± 0.06	1.20 ± 0.16	1.479 (0.742–2.948)
Asthma (*n*, %)	835 (9.66)	40 (20.7)	2.869 (1.866–4.410)
Allergies (*n*, %)	821 (9.84)	54 (11.0)	1.107 (0.794–1.541)
Psychological disease (*n*, %)	873 (9.93)	2 (4.88)	0.525 (0.070–3.947)

Values for continuous variables represented adjusted means ± standard error, and those for categorical variables represented the number of participants and percentage. Covariates: age, residence area, gender, BMI, job history in chemical and dust-related companies, smoking status, drinking status, total physical activity, medication for asthma, and energy intake. COPD definition: FEV1/FVC < 0.7 in spirometry. 95% CI, 95% confidence intervals. ^1^ cutoff: 0.7, ^2^ 55 years old, ^3^ 160 cm of height, ^4^ 23 kg/m^2^ for men and 22 kg/m^2^ for women, ^5^ 25% for men and 30% for women, ^6^ 8 × 10^3^/mL, ^7^ 0.5 mg/dL, ^8^ 5.4 μg/mL, ^9^ 1.5 μg/mL. * Significantly different between the Health and COPD groups at *p* < 0.05, ** at *p* < 0.01, and *** at *p* < 0.001. ^+++^ Significantly different among age groups at *p* < 0.001.

**Table 2:** Adjusted means of lifestyle, including nutrient intake between Health and COPD groups.

	Health (*n* = 7965)	COPD (*n* = 875)	Adjusted OR and 95% CI
Energy (EER%) ^1^	101 ± 0.43	102 ± 1.33	1.072 (0.900–1.276)
Carbohydrate (En%) ^2^	70.7 ± 0.07	70.5 ± 0.23	1.133 (0.920–1.394)
Protein (En%) ^3^	13.6 ± 0.03	13.7 ± 0.08	0.893 (0.744–1.073)
Fat (En%) ^4^	14.4 ± 0.06	14.6 ± 0.17	0.876 (0.728–1.053)
w-3 fatty acid (En%) ^5^	0.51 ± 0.01	0.50 ± 0.03	0.935 (0.790–1.106)
w-6 fatty acid (En%) ^6^	3.36 ± 0.02	3.36 ± 0.06	1.018 (0.858–1.206)
Fiber (g/day) ^7^	15 ± 0.06	15 ± 0.2	0.948 (0.764–1.175)
Total polyphenol (mg/day) ^8^	2060 ± 1.96	2078 ± 39.79	0.924 (0.742–1.151)
Total flavonoids (mg/day) ^8^	1459 ± 5.22	1467 ± 16.03	0.982 (0.784–1.229)
V-C (mg/day) ^9^	15 ± 0.06	15 ± 0.2	0.947 (0.789–1.137)
V-E (mg/day) ^9^	6.25 ± 0.04	6.3 ± 0.12	1.007 (0.819–1.240)
V-D (μg/day) ^9^	5.95 ± 0.06	6.27 ± 0.18	1.135 (0.905–1.424)
Se (μg/day) ^9^	29.8 ± 0.27	28.8 ± 0.83	0.865 (0.707–1.059)
Ca (mg/day) ^10^	475 ± 2.24	482 ± 6.91	1.039 (0.850–1.271)
Alcohol (g/day) ^11^	10.1 ± 0.24	9.18 ± 0.74	0.896 (0.708–1.133)
Non-smoking (*n*, %)	4753 (60.5)	389 (45.1) ***	1
Former smoking	1200 (15.3)	153 (17.8)	1.192 (0.874–1.625)
Current smoking	1908 (24.3)	320 (37.1)	1.581 (1.195–2.091)
Exercise ^12^	2168 (28.3)	312 (37.6) ***	0.980 (0.780–1.231)

Values for continuous variables represented adjusted means ± standard error, and those for categorical variables represented the number of participants and percentage. Covariates: age, residence area, gender, BMI, job history in chemical and dust-related companies, smoking status, drinking status, total physical activity, and energy intake. The cutoff of each parameter: ^1^ 100% of estimated energy requirement (EER), ^2^ 70 energy percent (En%) of carbohydrates, ^3^ 15 En% of protein, ^4^ 15 En% of fat, ^5^ 0.7 En% of w-3 fatty acid, ^6^ 4.5 En% of w-3 fatty acid, ^7^ 20 g/day of fiber, ^8^ 75th percentile of each intake, ^9^ DRI of each nutrient (100 mg for V-C, 10 mg for V-E, 10 ug for V-D, and 50 ug for Se), ^10^ 500 mg/day of Ca, ^11^ 20 g/day of alcohol, ^12^ 150 min/week of moderate exercise. *** Significantly different from the Health group.

**Table 3:** The characteristics of the ten genetic variants in COPD used for the generalized multifactor dimensionality reduction analysis.

Chr ^1^	SNP ^2^	Position	Mi ^3^	Ma ^4^	OR ^5^	SE ^6^	*p* Value for OR ^7^	MAF ^8^	*p* for HWE ^9^	Gene Names	Location
3	rs117262613	21603388	A	G	2.209	0.1958	5.16 × 10^−6^	0.0134	0.207	*ZNF385D*	NMD transcript variant
4	rs1585258	89879196	G	T	0.7895	0.0588	5.74 × 10^−6^	0.4271	1.0	*FAM13A*	NMD transcript variant
5	rs889294	52110676	A	G	1.281	0.0593	2.96 × 10^−5^	0.3251	0.191	*ITGA1*	NMD transcript variant
7	rs4145714	48391642	C	G	0.7009	0.0808	6.68 × 10^−7^	0.3653	0.277	*ABCA13*	intron variant
7	rs1997571	116198621	G	A	1.269	0.0598	4.96 × 10^−5^	0.3372	0.175	*CAV1*	NMD transcript variant
9	rs10959052	10332654	C	T	0.7293	0.0856	5.45 × 10^−7^	0.1456	0.266	*PTPRD*	Intron variant
11	rs74433025	132172642	C	T	2.156	0.1968	9.51 × 10^−6^	0.0140	1.0	*NTM*	Intron variant
17	rs719601	28731415	G	A	1.28	0.0663	2.12 × 10^−5^	0.2168	0.778	*CPD*	Intron variant
18	rs17482826	8796149	T	A	2.304	0.1676	6.41 × 10^−7^	0.0742	0.315	*MTCL1*	Intron variant
19	rs17569	33882222	A	G	1.251	0.0636	4.35 × 10^−5^	0.2433	0.112	*PEPD*	Missense p.His377Gln

^1^ Chromosome; ^2^ Single nucleotide polymorphism; ^3^ Minor allele; ^4^ Major allele; ^5^ Adjusted odds ratio; ^6^ Standard error; ^7^ *p*-value for OR adjusting for age, gender, residence area, education, body mass index, job history working in the chemical and dust related company, daily energy intake, alcohol intake, smoking, medication for asthma, and exercise; ^8^ Minor allele frequency; ^9^ Hardy–Weinberg equilibrium.

**Table 4:** Gene expression of COPD-related genetic variants in different tissues.

	Minor Allele	Beta Value	*p* Value	Tissues
*FAM13A_*rs1585258	G	−0.11	0.0055	Lung
*FAM13A_*rs1585258	G	−0.087	0.0093	Tibial nerve
*ITGA1*_rs889294	A	0.17	2.8 × 10^−10^	Skeletal muscle
*ITGA1*_rs889294	A	0.11	6.5 × 10^−6^	Subcutaneous adipose tissue
*ITGA1*_rs889294	A	0.13	8.4 × 10^−6^	Visceral adipose tissues
*ITGA1*_rs889294	A	0.11	0.00021	Tibial nerve
*ITGA1*_rs889294	A	0.11	0.00033	Left ventricle of the heart
*CAV1*_rs1997571	G	−0.078	0.000068	Tibial nerve
*CAV1*_rs1997571	G	0.13	0.0000035	Atrial appendage of the heart
*CPD*_rs719601	G	−0.089	0.0000026	Skeletal muscle
*CPD*_rs719601	G	−0.054	0.025	Lung
*PEPD*_rs17569	A	−0.39	6.9 × 10^−16^	Subcutaneous adipose tissue
*PEPD*_rs17569	A	−0.26	8.6 × 10^−10^	Visceral adipose tissues
*PEPD*_rs17569	A	−0.45	7.6 × 10^−6^	The cortex of the brain
*PEPD*_rs17569	A	−0.38	5.9 × 10^−16^	Skeletal muscle
*PEPD*_rs17569	A	−0.2	3.4 × 10^−9^	Tibial nerve
*PEPD*_rs17569	A	−0.28	5.3 × 10^−8^	Atrial appendage of the heart
*PEPD*_rs17569	A	−0.27	1.3 × 10^−6^	Left ventricle of the heart
*PEPD*_rs17569	A	−0.22	1.8 × 10^−14^	Lung

**Table 5:** Adjusted odds ratios (ORs) for the COPD risk by polygenic risk scores (PRS) of the 5-SNP model for genetic variant-lifestyle after covariate adjustments according to lifestyle patterns.

	Low-PRS (*n* = 2230)	Meddle-PRS *(n* = 4438)	High-PRS (*n* = 2172)	*p* Value for Interaction with PRS
Low-EER ^1^ High-EER	1 1	1.189 (0.912–1.549) 1.376 (1.000–1.892)	1.574 (1.179–2.101) 2.097 (1.487 2.956)	0.6221
Low-CHO ^2^ High-CHO	1 1	1.260 (1.002–1.584) 1.330 (0.852–2.074)	1.744 (1.360–2.236) 2.072 (1.278–3.361)	0.8662
Low-Protein ^3^ High-Protein	1 1	1.849 (1.488–2.296) 1.159 (0.847–1.586)	2.005 (1.419–2.832) 1.549 (1.100–2.180)	0.6349
Low-Fat ^4^ High-Fat	1 1	1.284 (0.948–1.740) 1.265 (0.961–1.664)	1.747 (1.296–2.355) 1.937 (1.356–2.769)	0.9926
Low-w3 fat ^5^ High-w3 fat	1 1	1.408 (1.031–1.924) 1.173 (0.900–1.530)	2.291 (1.642–3.197) 1.445 (1.076–1.941)	0.0183
Low-Fiber ^6^ High-Fiber	1 1	1.257 (1.000–1.579) 1.292 (0.823–2.026)	1.786 (1.392–2.291) 1.789 (1.104–2.888)	0.1119
Low-V-C ^7^ High-V-C	1 1	1.209 (0.910–1.605) 1.319 (0.984–1.767)	1.689 (1.238–2.304) 1.860 (1.357–2.550)	0.8173
Low-V-D ^8^ High-V-D	1 1	1.268 (1.008–1.595) 1.274 (0.821–1.977)	1.853 (1.447–2.373) 1.511 (0.923–2.472)	0.9739
Low-TP ^9^ High-TP	1 1	1.237 (0.823–1.860) 1.282 (1.014–1.621)	1.609 (1.031–2.509) 1.856 (1.440–2.393)	0.6323
Low-coffee ^10^ High-coffee	1 1	1.354 (0.987–1.857) 1.196 (0.916–1.561)	1.898 (1.343–2.683) 1.677 (1.258–2.234)	0.1935
Low-alcohol ^11^ High-alcohol	1 1	1.139 (0.915–1.418) 2.157 (1.257–3.700)	1.637 (1.288–2.080) 2.727 (1.544–4.818)	0.3373
Low-Exercise ^12^ High-Exercise	1 1	1.192 (0.944–1.506) 1.514 (1.037–2.212)	1.555 (1.203–2.001) 2.368 (1.576–3.560)	0.0056
Non-smokers Smokers	1 1	1.273 (1.039–1.559) 1.282 (0.967–1.700)	1.777 (1.426–2.215) 1.838 (1.354–2.494)	0.8442

Values represent adjusted odd ratios and 95% confidence intervals. Covariates included age, sex, education, income, energy intake (percentage of estimated energy requirement), residence areas, daily activity, alcohol intake, medication for asthma, and smoking status. The PRS with 5 SNPs of the best GMDR model was divided into three categories according to the number of the risk alleles: when the number of risk alleles in the PRS was ≤4, 5–6, and ≥7 into Low-PRS, Middle-PRS, and High-PRS, respectively. The reference was the low-PRS. The cutoff of each parameter: ^1^ 100% of estimated energy requirement (EER), ^2^ 70 energy percent (En%) of carbohydrates, ^3^ 15 En% of protein, ^4^ 15 En% of fat, ^5^ 0.7 En% of w-3 fatty acid, ^6^ 20 g/day of fiber, ^7^ 100 mg/day of vitamin C, ^8^ 10 μg/day of vitamin D, ^9^ 3220 mg/day of total polyphenol (TP), ^10^ 1 cup/day of coffee, ^11^ 20 g/day of alcohol, and ^12^ 150 min/week of moderate exercise.

## Data Availability

The original data could be provided from the Korean Center for Disease Control and Prevention.

## Supplementary Materials

The following supporting information can be downloaded at: https://www.mdpi.com/article/10.3390/nu15133062/s1, Supplementary Table S1: Stress-related questionnaires. Supplementary Table S2: Generalized multifactor dimensionality reduction (GMDR) results of multi-locus interaction with genes related to COPD risk.

## Abbreviations

FEV1, forced expiratory volume in 1 s; FVC, forced vital capacity; GOLD, Global Initiative for Chronic Obstructive Lung Disease; COPD, Chronic obstructive pulmonary disease; BMI, body mass index; SQFFQ, semi-quantitative food frequency questionnaire; GWAS, Genome-wide association studies; GMDR, generalized multifactor dimensionality reduction; Q–Q, Quantile–Quantile; SNPs, single nucleotide polymorphisms; LD, linkage disequilibrium; PRS, polygenic risk score; CVC, cross-validation consistency; TRBA, trained balance accuracy; TEBA, test balance accuracy; ORs, odds ratios; CI, confidence interval; ANOVA, analysis of variance; GLM, general linear model; DRI, dietary reference intake; CRP, C-reactive protein.
