# Supplementary material for: Impact of Lung-Related Polygenic Risk Scores on Chronic Obstructive Pulmonary Disease Risk and Their Interaction with w-3 Fatty Acid Intake in Middle-Aged and Elderly Individuals
Source: Nutrients. 2023 Jul 7;15(13):3062. doi: 10.3390/nu15133062 (PMC10346150; doi:10.3390/nu15133062)
Supplement: Supplementary file 1 [file nutrients-15-03062-s001.zip › nutrients-2494513-supplementary.pdf]

Supplementary Table S1. Stress-related questionnaires

The following 40 questionnaires to have the signs were answered for yes or no.

1. Stress-related behaviors for stress status
   - Symptoms: Tension, anger, nervous indigestion.
   - Triggers: People at work or home.
   - Coping mechanisms: Eating, drinking, smoking.
   - Symptoms: Tension, headaches, neck and shoulder pain, insomnia.
   - Relaxation during leisure time.
   - Difficulty concentrating on work due to worries.
   - Medication use for tension relief.
   - Limited time for relaxation.
   - Inability to use spare time for relaxation.
   - Rushed deadlines at work.

2. Psychological signs for stress status
   - Always anxious.
   - Easily gets excited or angry.
   - Decreased concentration and patience.
   - Severe absent-mindedness.
   - Prone to depression and feeling down.
   - Lack of motivation.
   - Excessive doubts and hesitation.
   - Low self-confidence and tendency to give up.
   - Inability to relax without doing something.
   - Impulsive judgments.

3. Physical signs for stress status
   - Shortness of breath.
   - Dry throat or mouth.
   - Insomnia.
   - Frequent headaches.

- Easily fatigued eyes.
- Shoulder and neck stiffness.
- Chest tightness and nausea.
- Decreased appetite.
- Constipation or diarrhea.
- Fatigue and sluggishness

4. Behavioral signs

- Increased arguments, complaints, and backtalk.
- More mistakes in work.
- Increased alcohol intake.
- Overly engrossed in work.
- Decreased speech and deep in thought.
- Increased talking and forceful opinions.
- Easily angered by trivial matters.
- Decreased interest in grooming and appearance.
- More personal calls or restroom visits at work.
- Increased absences, tardiness, or early departures (neglecting household chores or farming).

Supplementary Table S2. Generalized multifactor dimensionality reduction (GMDR) results of multi-locus interaction with genes related to COPD risk

	TRBA	TEBA	P value	CVC		TRBA	TEBA	P value	CVC
*CAV1*_rs1997571	0.5307	0.5022	0.623	5	*CAV1*_rs1997571	0.5329	0.5062	0.1719	5
*MTCL1*_rs17482826, *FAM13A*_rs1585258	0.5493	0.549	0.011	10	*MTCL1*_rs17482826, *FAM13A*_rs1585258	0.5475	0.5252	0.0107	6
*ITGA1*_rs889294, *CAV1*_rs1997571, *CPD*_rs719601	0.5591	0.5265	0.0547	5	*ITGA1*_rs889294, *CAV1*_rs1997571, *CPD*_rs719601	0.5613	0.5374	0.001	7
*FAM13A*_rs1585258, *CAV1*_rs1997571, *CPD*_rs719601, *PEPD*_rs10405598	0.5784	0.5177	0.377	4	*FAM13A*_rs1585258, *CAV1*_rs1997571, *CPD*_rs719601, *PEPD*_rs10405598	0.5785	0.5259	0.0547	5
*ITGA1*_rs889294 plus Model 4	0.6133	0.54	0.001	10	*ITGA1*_rs889294 plus Model 4	0.6109	0.543	0.001	10
*MTCL1*_rs17482826 plus Model 5	0.6503	0.5494	0.011	9	*MTCL1*_rs17482826 plus Model 5	0.6448	0.5352	0.001	7
*PTPRD*_rs10959052 plus Model 6	0.686	0.5461	0.001	10	*PTPRD*_rs10959052 plus Model 6	0.6805	0.5387	0.001	10
*ABCA13*_rs4145714 plus Model 7	0.7175	0.5443	0.001	10	*ABCA13*_rs4145714 plus Model 7	0.7132	0.5493	0.001	10
*ZNF385D*_rs117262613 plus Model 8	0.73	0.5401	0.001	9	*ZNF385D*_rs117262613 plus Model 8	0.7241	0.543	0.001	9
*NTM*_rs74433025 plus Model 9	0.7404	0.5438	0.001	10	*NTM*_rs74433025 plus Model 9	0.7337	0.5415	0.001	10

Adjusted for age, gender, BMI, education, and residence area for model 1. Adjusted for covariates for Model 1 plus energy intake, alcohol intake, smoking, medication for asthma, and exercise.
